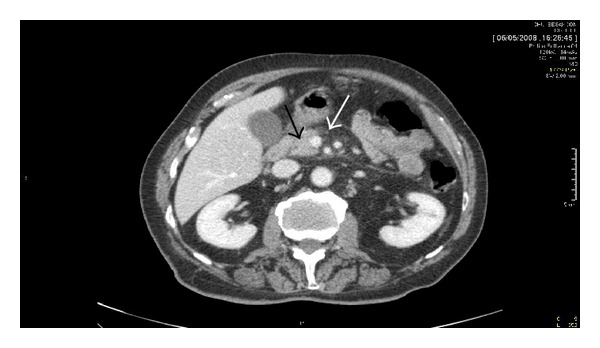# Acute Pancreatitis Occurring after Pamidronate Infusions in Two Patients with Spondyloarthritis

**DOI:** 10.1155/2013/912692

**Published:** 2013-06-13

**Authors:** Éric Toussirot, Lucine Vuitton, Stéphane Koch, Marie-Blanche Valnet-Rabier

**Affiliations:** ^1^Clinical Investigation Center in Biotherapy, CBT-506, University Hospital of Besançon, Bâtiment St Joseph, CHRU, 2 Place St Jacques, 25000 Besançon, France; ^2^Department of Rheumatology, University Hospital of Besançon, 25000 Besançon, France; ^3^Department of Therapeutics and UPRES EA 4266 “Pathogens and Inflammation”, University of Franche Comté, 25000 Besançon, France; ^4^Department of Gastroenterology, University Hospital of Besançon, 25000 Besançon, France; ^5^Department of Pharmacovigilance and Drug Information, University Hospital of Besançon, 25000 Besançon, France

## Abstract

We report two cases of acute pancreatitis following the administration of pamidronate given as an anti-inflammatory agent for spondyloarthritis with a recurrence in one patient when the drug was reintroduced. The upper gastrointestinal toxicity of aminobisphosphonates is well known and this drug class could be added to the list of medications that are associated with the development of pancreatitis.

## 1. Introduction

Drugs are a relatively uncommon cause of acute pancreatitis (AP) [1, 2]. Pamidronate is an aminobisphosphonate (ABPP) with anti-inflammatory properties that is used in the treatment of spondyloarthritis [3]. We report here two cases of such patients who developed AP while receiving pamidronate.

## 2. Case One

**Figure 1:**
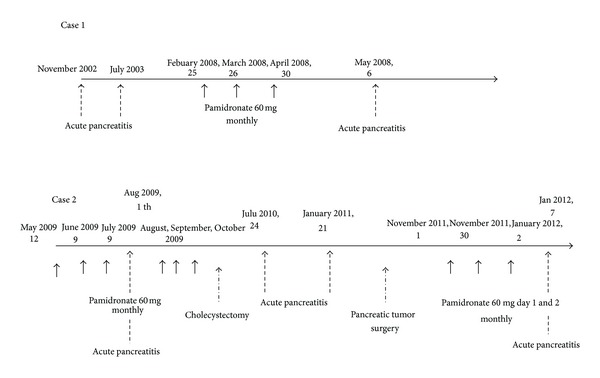


A 70-year-old white male with a 40-year history of ankylosing spondylitis (AS) was referred to our hospital for abdominal pain. His AS had been treated previously by different NSAIDs with a progressive lack of efficacy. He had a past medical history of dyslipidemia treated by fenofibrate for more than ten years, two episodes of AP without any identified cause, and prostate cancer, a contraindication to use anti-TNF*α* therapy. He was treated for active AS by infusions of pamidronate 60 mg monthly for three months. Ten days after the third infusion, he presented with abdominal pain radiating to the back without nausea or vomiting (Figure 1). The laboratory data at admission were unremarkable without elevated serum lipase levels (evaluated one week after the first abdominal symptoms). He had neither hypercalcemia nor hypertriglyceridemia. A CT scan of the abdomen with intravenous contrast showed edema of pancreatic and peripancreatic tissue that was consistent with AP (computed tomography score index-CTSI-2) (Figure 2). An ultrasound endoscopy and magnetic resonance cholangiopancreatography showed a normal biliary tract without lithiasis. The patient denied alcohol abuse and had never been diagnosed with inflammatory bowel disease (IBD). He improved promptly after bowel rest and parenteral pain management. Pamidronate was not reintroduced.

## 3. Case Two

 The second patient was a 39-year-old white female with peripheral arthritis and inflammatory back pain related to spondyloarthritis. She received NSAIDs, sulfasalazine, and methotrexate successively, all of which were ineffective. She developed infections under adalimumab. Due to persistent back pain, pamidronate was started in August 2009. She received six pamidronate infusions, 60 mg monthly, leading to progressive improvement. Three weeks after the third infusion, she complained of abdominal pain with elevated serum lipase (184 UI/L; N < 60) and AP was seen on injected CT scan of the abdomen (CTSI 2). Microlithiasis was observed on abdominal ultrasound and the patient thus underwent laparoscopic cholecystectomy surgery. She had two other episodes of AP without receiving pamidronate and a nonmalignant caudal pancreatic tumor was then diagnosed in 2010 (glucagonoma). This tumor was removed by surgery and pamidronate treatment was restarted in 2011. Five days after the third infusion, she again had AP diagnosed on a CT scan of the abdomen (Figure 1). This patient had neither dyslipidemia, nor IBD, nor a history of alcohol intake. PTH serum level was normal. The ABPP was permanently withdrawn. No new episode of AP after pamidronate cessation was observed in either of these two patients. 

The safety profile of pamidronate is well described, with acute-phase reaction following its administration but no evident abdominal organ toxicity [4]. Drug-induced pancreatitis is a rare cause of AP, estimated at between 0.1 and 2% of all AP [1, 2]. Most of the information available on drug-induced AP comes from single case reports with varying levels of evidence [5]. Our two patients had no predisposing factors for AP and the other usual causes of AP were excluded (alcoholism, hypertriglyceridemia, hypercalcemia, IBD, trauma, and surgery). The first patient had a past history of AP with no identified cause while the second had microlithiasis and a pancreatic tumor, but despite cholecystectomy and pancreas surgery, she experienced another episode of AP after pamidronate was reintroduced. In these two cases, there was a clear temporal relationship between the pamidronate infusions and the development of AP (ten days for the first patient, three weeks and five days for the second). In the second case, a rechallenge of the drug caused a recurrence of AP. According to the Badalov classification of drugs that induce AP, pamidronate may be considered as a class Ib drug with a high level of causality [6]. Our two patients scored +5 and +7, respectively, on the Naranjo probability scale for drug causality assessment [7]. One case of suspected pancreatitis has been reported with alendronate, also with a possible causality [8]. ABPPs do not diffuse specifically to the pancreas and a direct toxic effect thus seems unlikely. Our two patients had no systemic reaction and a hypersensitivity reaction was therefore not evident. Conversely, ABPPs can modulate the immune system and the production of proinflammatory cytokines [3]. In a mouse model of induced pancreatitis, macrophage ablation by liposome encapsulated bisphosphonates may worsen pancreatic inflammation [9]. However, our two patients developed other flares of AP without concomitant pamidronate infusion and we cannot therefore exclude the hypothesis of a spontaneous recurrence for the episodes following pamidronate infusions. Alternatively, pamidronate may have played a role as a triggering factor in these patients with previous history of pancreatic damage and thus made a new recurrence more likely. The mechanisms explaining drug-induced pancreatitis are in general poorly understood [1] and collecting data about all drug-induced pancreatitis, including ABPPs, should help to better identify the contributing factors of such an adverse event. 

## Figures and Tables

**Figure 2:**